# Dual-energy computed tomography and micro-computed tomography for assessing bone regeneration in a rabbit tibia model

**DOI:** 10.1038/s41598-024-56199-8

**Published:** 2024-03-12

**Authors:** Danyang Su, Yan Wu, Shenyu Yang, Duoshan Ma, Haoran Zhang, Yuanbo Ma, Jinlong Liu, Chunyu Wang, Huilong Liu, Xiaopeng Yang

**Affiliations:** 1 Department of Radiology, The First Affiliated Hospital of Zhengzhou University, Zhengzhou, China; 2 Department of 3D Printing Center, The First Affiliated Hospital of Zhengzhou University, Zhengzhou, China; 3 Department of Medical Equipment, The First Affiliated Hospital of Zhengzhou University, Zhengzhou, China

**Keywords:** DECT, Micro-CT, Bone regeneration, Porous PEEK implants, Calcium density, Hydroxyapatite density, Biochemistry, Biological techniques, Endocrinology, Medical research, Energy science and technology, Engineering, Materials science

## Abstract

To gain a more meaningful understanding of bone regeneration, it is essential to select an appropriate assessment method. Micro-computed tomography (Micro-CT) is widely used for bone regeneration because it provides a substantially higher spatial resolution. Dual-energy computed tomography (DECT) ensure shorter scan time and lower radiation doses during quantitative evaluation. Therefore, in this study, DECT and Micro-CT were used to evaluate bone regeneration. We created 18 defects in the tibial plateau of the rabbits and filled them with porous polyetheretherketone implants to promote bone regeneration. At 4, 8, and 12 weeks, Micro-CT and DECT were used to assess the bone repair in the defect region. In comparison to Micro-CT (152 ± 54 mg/cm^3^), the calcium density values and hydroxyapatite density values obtained by DECT [DECT(Ca) and DECT(HAP)] consistently achieved lower values (59 ± 25 mg/cm^3^, 126 ± 53 mg/cm^3^). In addition, there was a good association between DECT and Micro-CT (R = 0.98; R^2^ = 0.96; DECT(Ca): y = 0.45x–8.31; DECT(HAP): y = 0.95x–17.60). This study highlights the need to use two different imaging methods, each with its advantages and disadvantages, to better understand the bone regeneration process.

## Introduction

Tibial plateau fracturs are common intra-articular fracture in clinical practice. In collapsed fractures, large bone defects are often observed after reduction. Without effective bone graft packing and fixation, weight-bearing walking can lead to recollapse and displacement. Rabbits are a good animal model for testing the repair of tibial plateau bone defects because their joints are sufficiently large to perform simple surgical procedures^1^.

Disease models that closely mimic human conditions must be developed to obtain a meaningful understanding of bone regeneration in humans. Ideally, disease models and study designs should closely reflect the pathology of patients with bone defect. Therefore, the methodologies used to diagnose, monitor disease progression, and assess treatment effects should be transferred to clinical settings. However, researchers using animals in preclinical studies have developed several techniques that cannot be replicated in the clinical setting. For example, histological techniques and micro-computed tomography (Micro-CT). The majority of research employs Micro-CT to generate accurate and comprehensive bone regeneration data for the quantification of bone microarchitecture, improving the estimation of bone quantity^2–11^. However, new assessment techniques are required because of issues with extended scanning times and high radiation doses. The rapid improvement in CT, which has significantly enhanced the imaging speed and resolution, has led to an increase in the number of researchers seeking to employ clinical CT to creat three-dimensional (3D) images of bone in experimental animals and humans^2–4,6,7,9–11^. In addition, the arrival of dual energy computed tomography (DECT) has provided new hope for visualization and more accurate quantitative analyses. Although the resolution of DECT is lower than that of Micro-CT, the material decomposition technology of DECT has the advantage of precisely assessing the density of calcium (Ca) or hydroxyapatite (HAP) because it models bone signals in terms of bone mineral^4,12–14^.

Bissinger et al. used bone defect specimens to demonstrate that multirow detector CT (MDCT) is sufficient to assess bone repair in comparison with Micro-CT^4^. Diederichs et al. found significant relationships between the measurements of the bone volume fraction obtained by Micro-CT and MDCT^6^. The accuracy and consistency of volumetric bone mineral density (BMD) measurements made using Micro-CT and DECT were tested by Mussmann et al.^15^, who also asserted that employing virtual monochromatic DECT imaging can contribute to research on bone loss near hip replacements. However, no studies have examined bone defect repair using DECT material decomposition techniques and using a reference standard such as Micro-CT.

The primary indicator of the amount of bone is volumetric BMD^13,16,17^, which has proven useful for treatment monitoring. The repair of bone defects remains challenging in clinical setting^18,19^ and it is crucial to find an appropriate technique for assessing bone regeneration. Polyetheretherketone (PEEK), a new type of bone defect-filling material, has been widely used in many clinical fields. Therefore, we aimed to use rabbit defect models of the tibial plateau to investigate bone regeneration by using DECT and Micro-CT (Table 1).

**Table 1:** Study setup indicating the time points for death and the corresponding numbers of specimens.

Week	4	8	12
Number of rabbit tibia specimens	6	6	6

## Results

**Figure 1:**
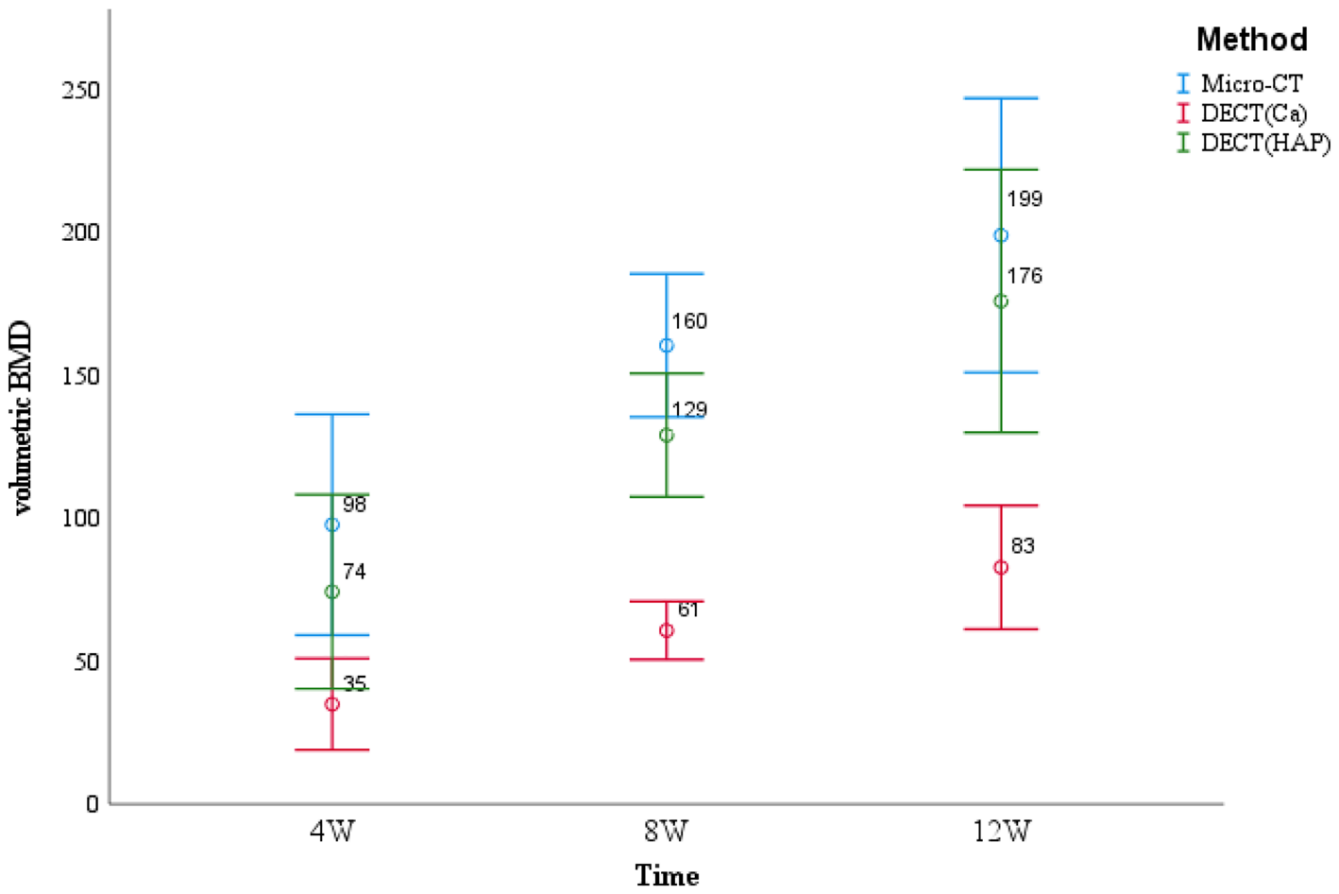
DECT(Ca), DECT(HAP) and micro-CT were used to obtain the trend of volumetric BMD values in different periods.

Compared with Micro-CT, DECT(Ca) and DECT(HAP) significantly underestimated the volumetric BMD values. DECT(Ca) refers to the calcium density value obtained from the calcium-water based material pair in DECT,and DECT(HAP) refers to the hydroxyapatite density value obtained using hydroxyapatite-water based material pairs in DECT. The average difference between DECT(Ca) and Micro-CT measurements was 93 mg/cm^3^, whereas that between DECT(HAP) and Micro-CT measurements was 26 mg/cm^3^ (Table 2). In addition, it is not difficult to find that the change trend of volumetric BMD values obtained by DECT(HAP) and Micro-CT in different periods is more similar than DECT(Ca) (Fig. 1).

**Table 2:** Mean, standard deviation, and range of bone mineral density for DECT and Micro-CT, with the *p*-value of the comparison between the two techniques.

	DECT(Ca) (mg/cm^3^)	DECT(HAP) (mg/cm^3^)	Micro-CT (mg/cm^3^)
Mean	59	126	152
Standard deviation	25	53	54
Range	[24–103]	[50–219]	[70–252]
P-value	*p* < 0.001; F = 19.156

**Figure 2:**
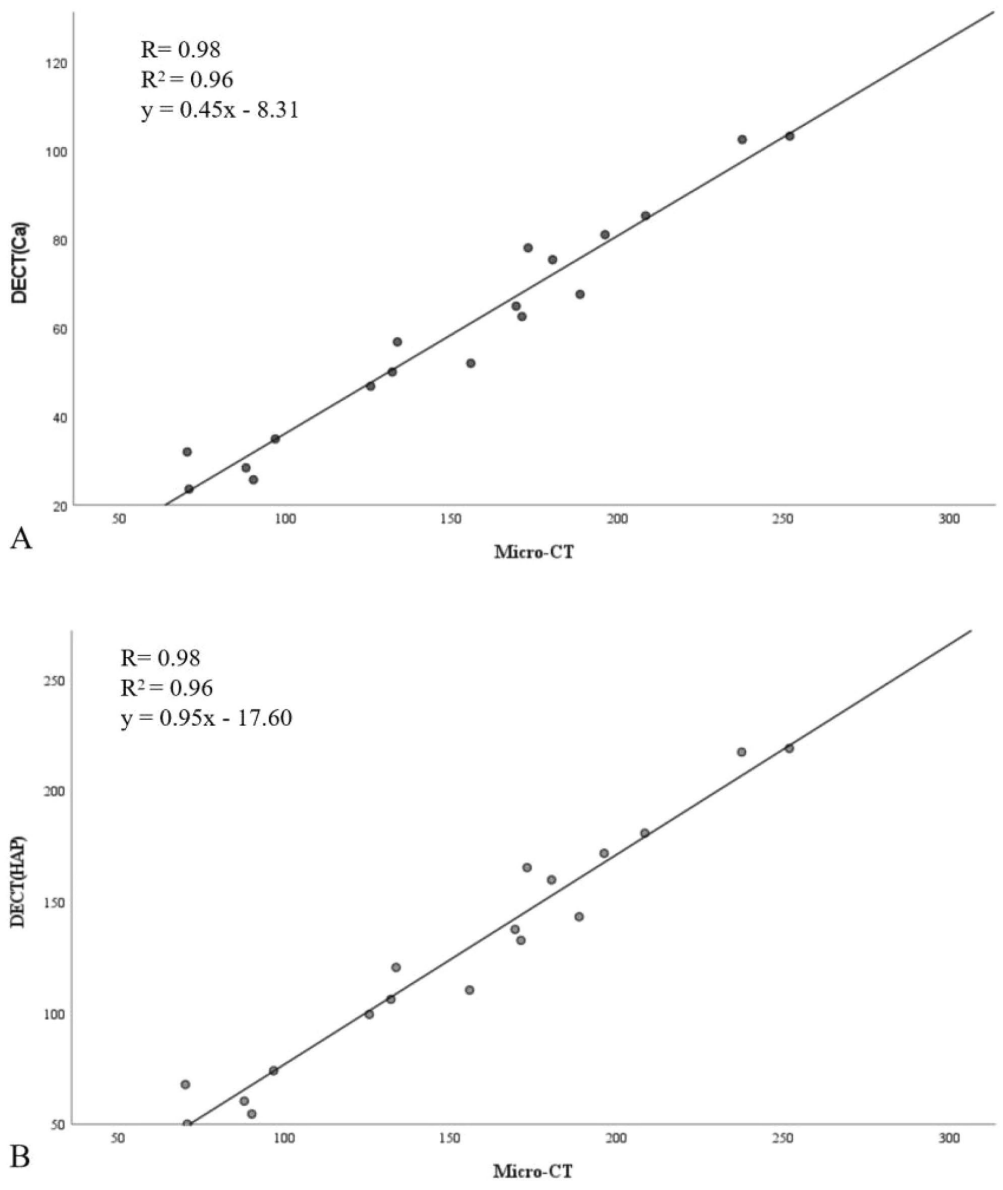
Scatter plots with a regression line, correlation coefficient (R), coefficient of determination(R^2^) and estimated regression model showing the associations between volumetric BMD obtained via Micro-CT and DECT(Ca) (**A**), as well as volumetric BMD obtained via Micro-CT and DECT(HAP) (**B**).

There were significant correlations between DECT(Ca) and Micro-CT (R = 0.98, R^2^ = 0.96, Fig. 2), as well as between DECT(HAP) and Micro-CT (R = 0.98, R^2^ = 0.96, Fig. 2). The regression equation for Micro-CT and DECT(Ca) was y = 0.45x–8.31 (Fig. 2), while for Micro-CT and DECT(HAP), it was y = 0.95x-17.60 (Fig. 2). In summary, DECT consistently generated lower volumetric BMD values, which were strongly correlated with Micro-CT.

**Figure 3:**
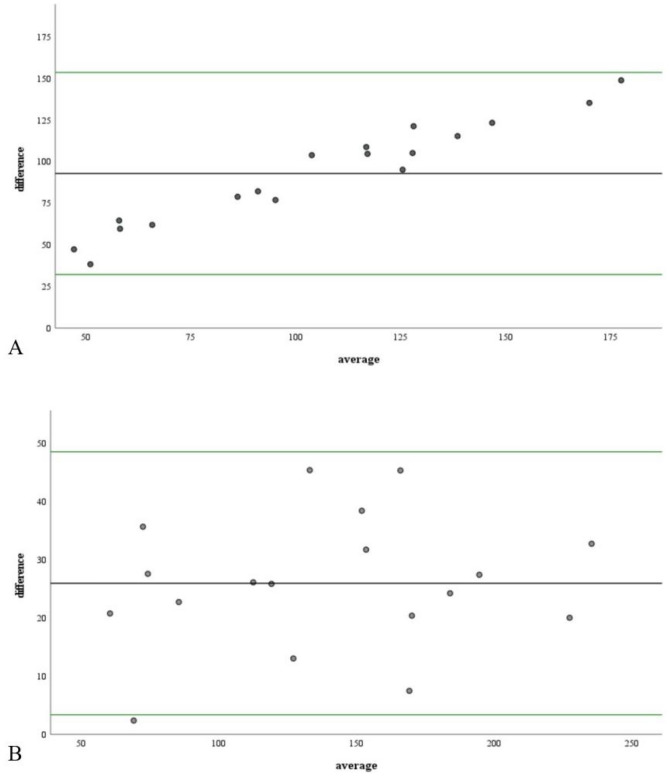
Bland–Altman plot of the difference between the measurements obtained by the two methods against their mean value. (**A**) is for DECT(Ca), and (**B**) is for DECT(HAP). The black line displays the mean difference, representing the bias between the methods. The green lines indicate the limits of agreement (mean of the differences ± 1.96 SD of the differences).

The bias, which indicates the systematic error, is represented in the Bland–Altman plot (Fig. 3) as the mean difference (presented by a black line, deviating from 0) between measurements acquired using DECT and Micro-CT. For the two base materials, most of the differences lay between the mean of the differences ± 1.96 SD (illustrated by the green lines), which indicated high agreement between both measurements. It is believed that the area outside the green line is the result of irregularities in the delineation of the area and will require additional regulation in the future.

**Figure 4:**
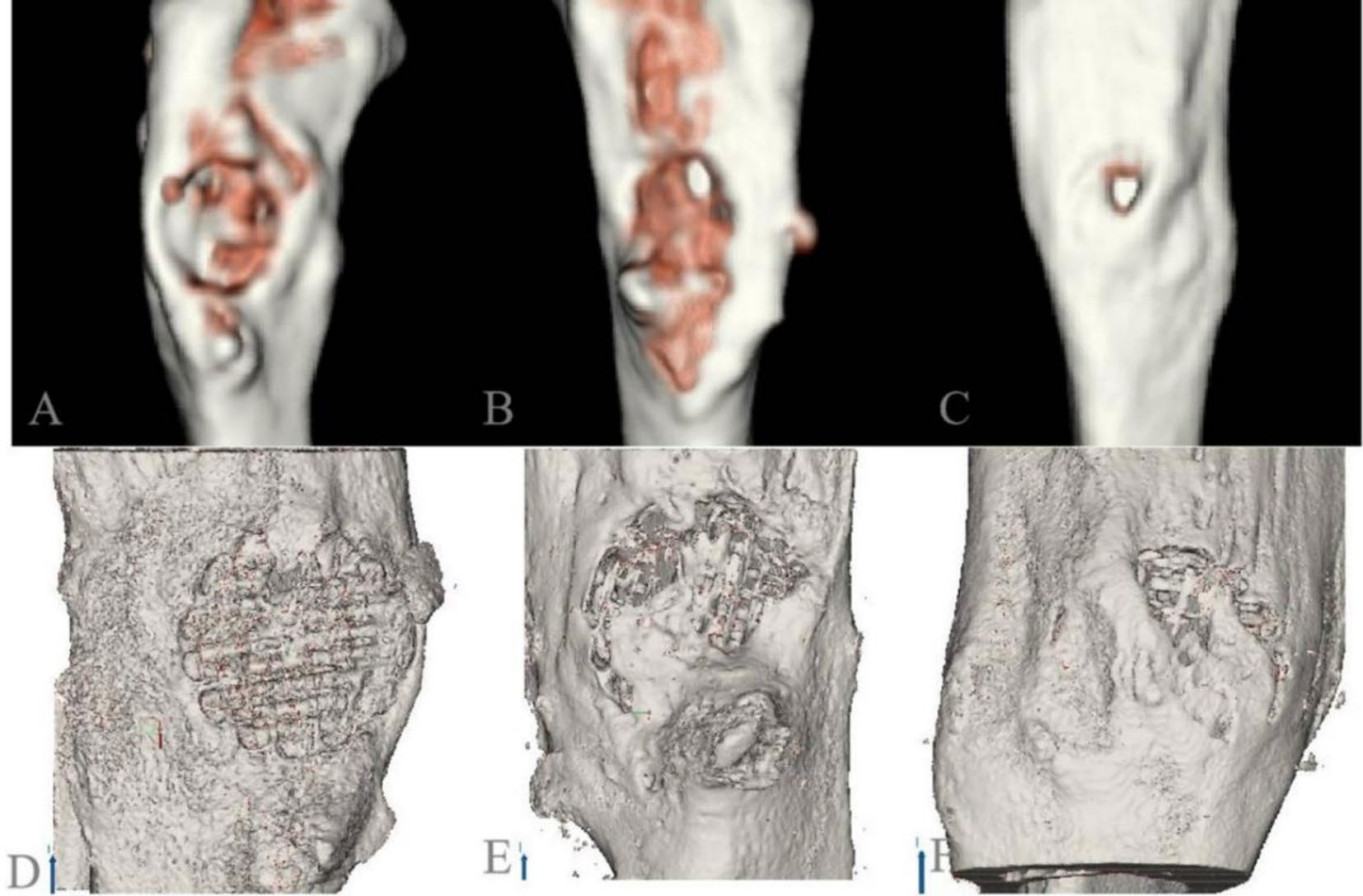
3D imaging from DECT (**A**–**C**) and micro-CT (**D**–**F**) scans after post-processing. In images (**A**–**C**), the color of the bone defect area gradually changed from gold to white, indicating that the bone repair effect was better. In the three images (**D**–**F**), the bone appeared gray, and the more grayer deposition, the better the bone repair effect.

Finally, we observed the degree of bone repair by three-dimensional reconstruction of the specimens (Fig. 4). In images A, B, and C, the color of the bone-defect area gradually changed from gold to white, indicating a better bone-repair effect. In images D, E, and F, the bone appears gray, and the more gray the deposition, the better the bone repair effect. We can observe that DECT is inferior to 3D Micro-CT for evaluating bone repair. Although the resolution of DECT is not as excellent as that of Micro-CT, by using diverse colors in 3D images, DECT images can show how the degree of bone repair varies in different areas.

## Discussion

This study aimed to examining the repair for tibial plateau bone defects in New Zealand rabbits using DECT material decomposition techniques and a reference standard such as Micro-CT. Micro-CT serves as the reference standard for assessing bone structure because of its high resolution. CT is a recognized modality for measuring bone mineral content and stands out for its rapid scan speeds and lack of invasiveness^2,20,21^. In recent years, the material decomposition of DECT has created new opportunities for the quantitative assessment of different tissues or components^12,14,22^. In conclusion, we observed that DECT is adequate for determining the evolution of bone regeneration.

More specifically, over time, DECT consistently produced lower volumetric BMD values than the equivalent Micro-CT data. Owingto the lower resolution and larger voxel sizes of the DECT images, partial volume effects were responsible for this conclusion^4^. Additionally, the mean density difference between Micro-CT and DECT(HAP) was lower than that between Micro-CT and DECT(Ca). DECT(HAP) exhibited a more consistent trend with Micro-CT (Fig. 1). This is because the measurements were more precise when HAP was used, which more closely resembles the actual composition of bone minerals than calcium^23^. By modeling bone signals in terms of bone mineral (Ca or HAP)^12–14^, which is possible with DECT, one may be able to precisely quantify volumetric BMD rather than simply relying on net attenuation. There is a highly significant correlation between these approaches. The deviation between DECT and Micro-CT was corrected using the relevant regression equation (Fig. 2). Based on this advantage, with only Micro-CT scanning in a limited number of samples, or ideally without Micro-CT scanning, future studies are expected to rely on DECT data for the assessment of bone repair.

The development of quantitative image processing methods for assessing volumetric BMD has been the focus of Micro-CT and clinical CT imaging^24–27^. Recently, DECT has been used to evaluate the volumetric BMD in dental imaging^28^. Simulation research has revealed an accurate estimation of volumetric BMD using DECT in musculoskeletal imaging^29^. Additionally, it has been demonstrated that DE-based measures of volumetric BMD in multidetector CT may be more accurate than single-energy quantitative CT^22,27,30^. The Bland–Altman plot indicated that 95% of the data points should lie within ± 1.96 SD of the mean difference (Fig. 3). This study demonstrates that volumetric BMD measured by DECT is also feasible in bone repair studies compared to Micro-CT. In addition, the volumetric BMD values of DECT measurements obtained after correction using regression equations can better reflect the repair of bone defects.

Micro-CT enables sequential in vivo measurements, and the distribution of new bone allowing scaffold observation in the 3D reconstruction map with high resolution (Fig. 4). However, the ionizing radiation that animals are exposed to progressively accumulates^5,31^. The Micro-CT dose was assumed to be higher than that of clinical CT^32^. By applying various pseudocolors and transparencies, 3D imaging using DECT revealed the distribution and degree of new bone repair (Fig. 4). Owing to the nature of our ex vivo investigation, our methods were not optimized for low radiation dosages in DECT or Micro-CT. More investigations are needed to optimize radiation exposure in in vivo Micro-CT analysis and DECT analysis^7,9,33^. We employed rabbit defect models to confirm the feasibility of our results because the model is thought to be economically favorable and the mechanics of fracture healing are similar^34^.

Our study had some limitations. First, we used specimens; however, the surrounding soft tissues must be considered for in vivo applications. Therefore, studies should be conducted to establish Micro-CT and DECT imaging in rabbits after the application of a scaffold in vivo. In addition, although the samples were carefully positioned, it was difficult to keep the position of the region of interest (ROI) consistent for each delineation, and even small changes may have an impact on the measured values. This may be the reason for some of the variation that still need to be improved in the future. DECT holds great promise for calculating volumetric BMD, but further research is necessary to identify the coefficient of variation. Daqian et al. utilized DECT technology as a beneficial tool for bone defect treatment by employing real-time and noninvasive cell tracking^35^. In the future, the accuracy and reliability of bone healing assessment will be greatly improved if DECT can be used in conjunction with cells and bone for monitoring.

In summary, this article addresses the issue of the lack of nonnvasive, real-time tracking of bone mineral signals with the clinical DECT technique for the first time and provides a practical tool for further bone repair research compared with Micro-CT. Owing to the noninvasiveness, quick scan times, accuracy in collecting volumetric BMD, and low radiation of DECT based on our model, consecutive in vivo scans appear to be practical and rational in the future. Future investigations of bone repair could save a considerable number of animals, time, and financial resources owing to the availability of DECT material decomposition technology.

In conclusion, the data from this study suggest that although the Micro-CT and DECT methods can be used as stand-alone techniques, their combinationadds value. Evaluation of bone regeneration requires the use of different approaches to adequately address all aspects of the disease. Owing to the availability of DECT material decomposition techniques, it is beneficial to research the effects of different defect sites and materials or strategies on bone regeneration, and which is of great significance to further promote the repair of human bone defects.

## Methods

All procedures and animal studies were approved by the the Experimental Animal Ethics Committee of Jinan University (No. 20210426-02). All procedures were performed in accordance with the relevant guidelines and regulations of the Guide for the Care and Use of Laboratory Animals published by the National Institutes of Health (NIH Publication No. 85-23). Eighteen tibial specimens from male New Zealand rabbits aged 4 months were randomly divided into three groups, with six rabbits in each group. The animals’ well-being was maintained throughout the experimen, adhering to the Experimental Animal Protection Law. The tibial plateau was surgically induced with an 8 mm defect, which was subsequently treated by grafting porous PEEK scaffolds (with a diameter of 8 mm and a depth of 3 mm) fabricated using Fused Deposition Modelling 3D printing technology (Jugao-AM Tech. Corp, Xi’ an, China). After 4, 8, and 12 weeks of implantation (group size per time point: 6), and the rabbits were euthanized by carbon dioxide asphyxiation, and the specimens were then removed and stored in a 10% formaldehyde solution.

The samples were scanned with a Micro-CT system (SkyScan 1272, Bruker, Germany), which had a voxel size of 20 × 20 × 20μm^3^, and the three-dimensional structure was reconstructed. The location of the samples were tightly set each time they were scanned. The energy and intensity settings for the Micro-CT were 85 kVp and 117 μA, respectively. With an exposure time of 100 ms per projection. Each specimen was examined for a total of approximately 40 min. A cylindrical volume of interest with a diameter of 8 mm and a thickness of 3 mm was precisely chosen in the bone defect area, and the volumetric BMD at different implantation times points were systematically analyzed. Volumetric BMD values obtained from Micro-CT were compared with those obtained using DECT (Supplementary Fig. 1).

**Figure 5:**
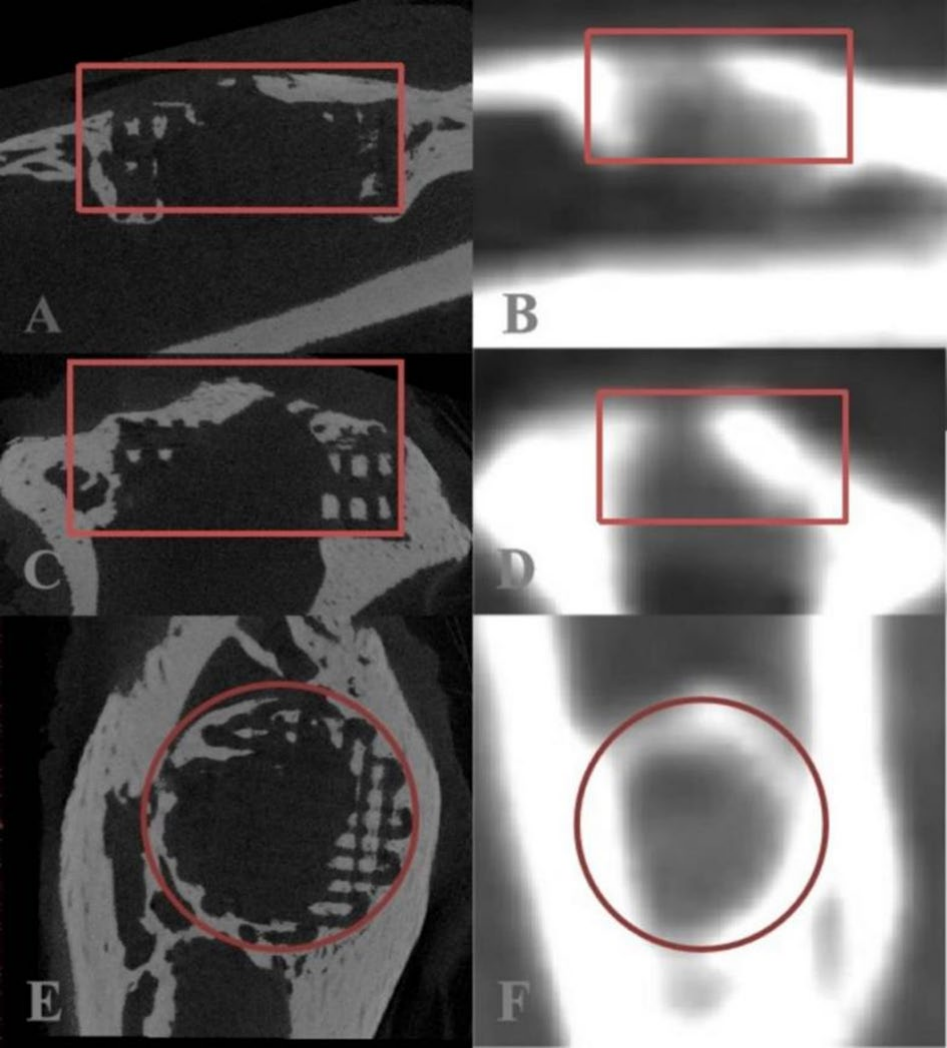
Micro-CT (**A**, **C**, and **E**) and DECT (**B**, **D**, and **F**) images of the defect region.

A KVSCT scanner (GE Healthcare, WI, USA) was used to gather images for the bone repair analysis, and the three-dimensional structure was reconstructed from the tomographic images. The scanning was initially performed in helical mode with a tube voltage of 80 kVp and 140 kVp instantaneous high-speed switch, tube current of 200 mA, pitch of 0.992:1, rotation time of 0.5 s, field of view (FOV) of 250 mm, a slice thickness and interval of 5.00 mm, reconstruction slice thickness and interval of 0.625 mm. DECT can actualize the scanning of numerous specimens simultaneously and can finish the scanning in a few seconds. The images were transferred to Advantage Workstation version 4.7 (GE Healthcare, WI, USA). At this workstation, the base material images of Ca (water) and HAP (water) were generated using a material decomposition analysis tool. Delineating the ROI made it possible to measure the DECT (Ca) and DECT (HAP) values, which were then compared with the values determined using Micro-CT. Because of the obvious line separating the defect from the surrounding bone in all modalities, a circle could be precisely placed to define the ROI (Fig. 5).

### Statistical analysis

SPSS (version 26.0) was used for all the statistical analyses. All data are displayed as mean values ± standard deviations ($\overline{{\text{x}} }\pm$ s). We used Pearson's correlation coefficient to investigated the correlation between the Micro-CT and DECT data. To further investigate the relationship between DECT and Micro-CT dada, a linear regression model was fitted using DECT dada as the dependent variables and the Micro-CT data as the independent variables. The agreement between the modalities was evaluated using Bland–Altman plots that display the difference in measurements versus the mean of the measurements^36^. For all tests, a *p-*value of less than 0.05 was considered significant.

### Ethics approval and consent to participate

All protocols in this study were approved by the Experimental Animal Ethics Committee of Jinan University (No. 20210426-02), in compliance with the Guide for the Care and Use of Laboratory Animals published by the US National Institutes of Health (NIH publication no.85-23). And we confirmed that the study in my manuscriptis was reported in accordance with ARRIVE guidelines.

### Supplementary Information

Supplementary Figure 1.

Supplementary Figure 2.

Supplementary Figure 2.

Supplementary Figure 2.

## Data Availability

The data that support the findings of this study are available from the corresponding author, upon reasonable request.
