# Supplementary figures and images for: Dual-energy computed tomography and micro-computed tomography for assessing bone regeneration in a rabbit tibia model
Source: Sci Rep. 2024 Mar 12;14:5967. doi: 10.1038/s41598-024-56199-8 (PMC10933353; doi:10.1038/s41598-024-56199-8)

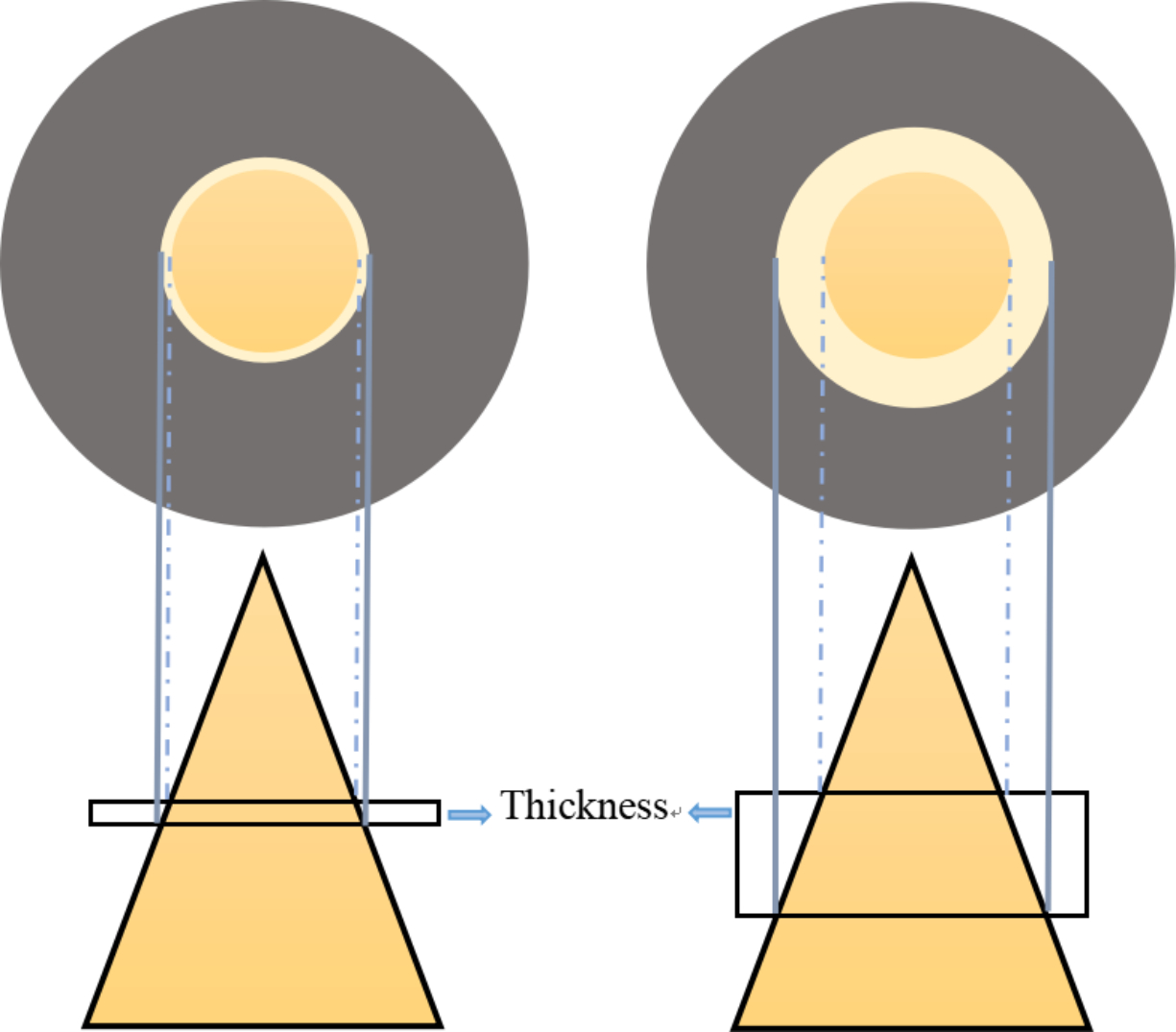

Supplement: Supplementary file 1 — Supplementary Figure 1. [file 41598_2024_56199_MOESM1_ESM.jpg]

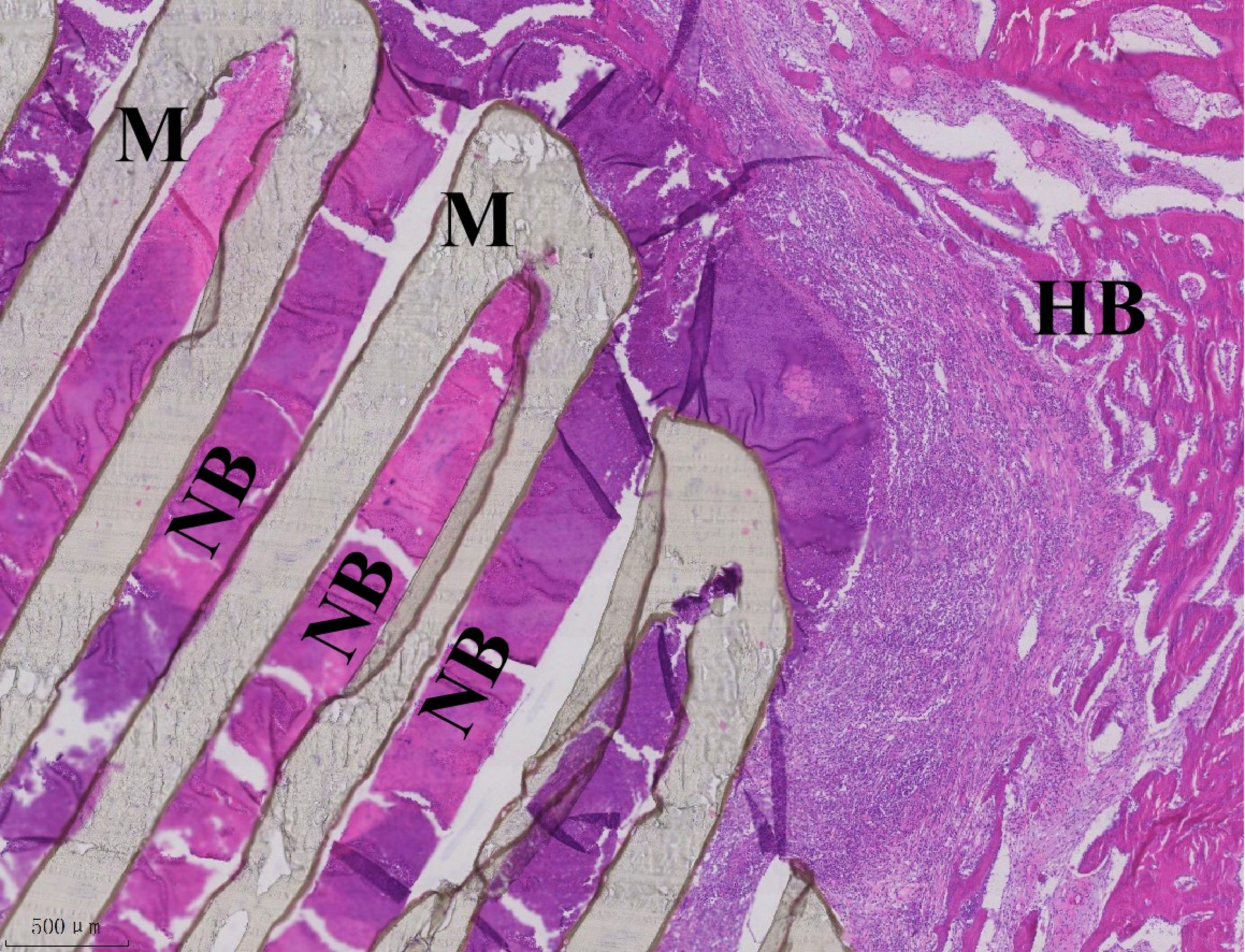

Supplement: Supplementary file 2 — Supplementary Figure 2. [file 41598_2024_56199_MOESM2_ESM.jpg]

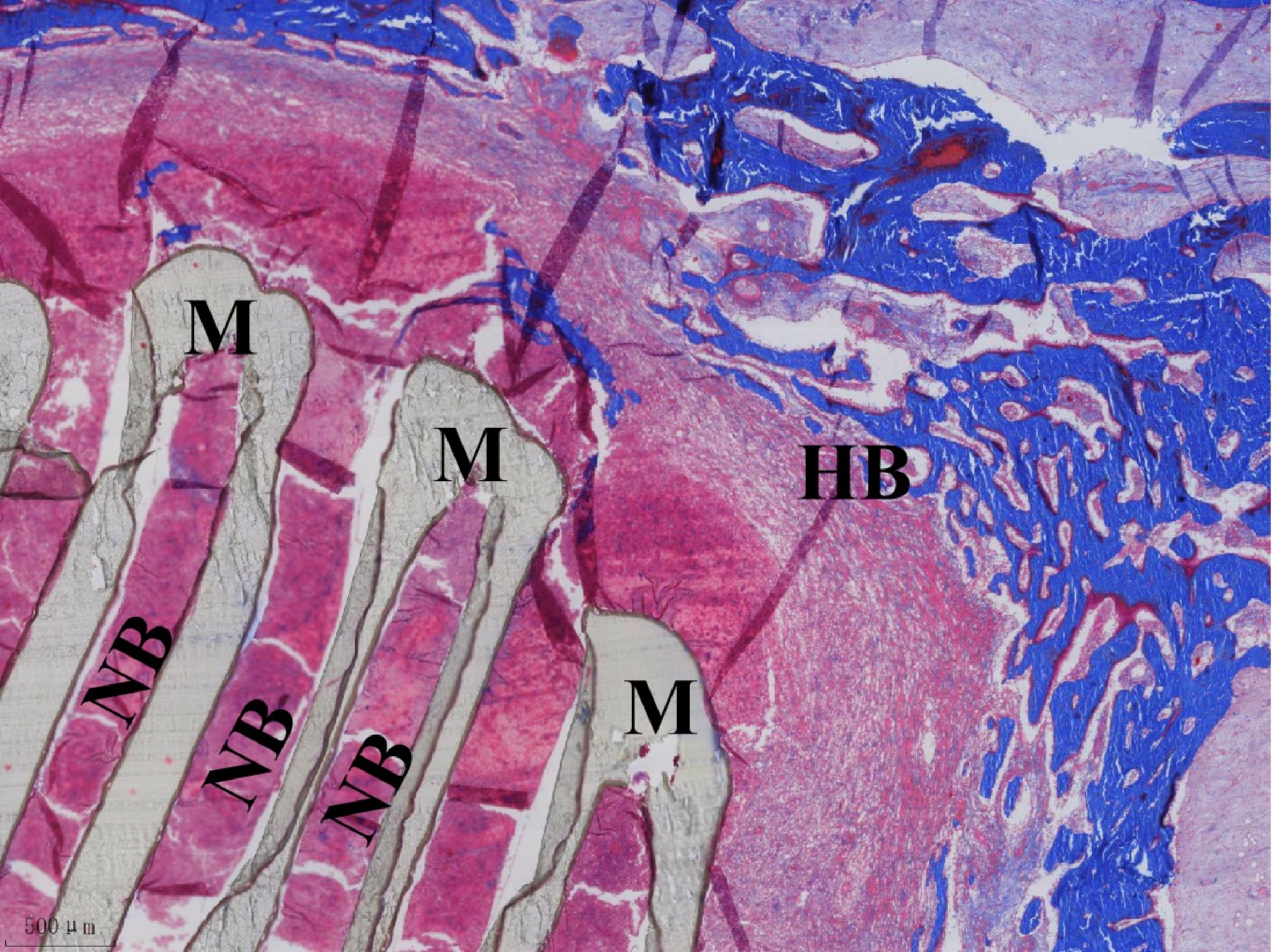

Supplement: Supplementary file 3 — Supplementary Figure 2. [file 41598_2024_56199_MOESM3_ESM.jpg]

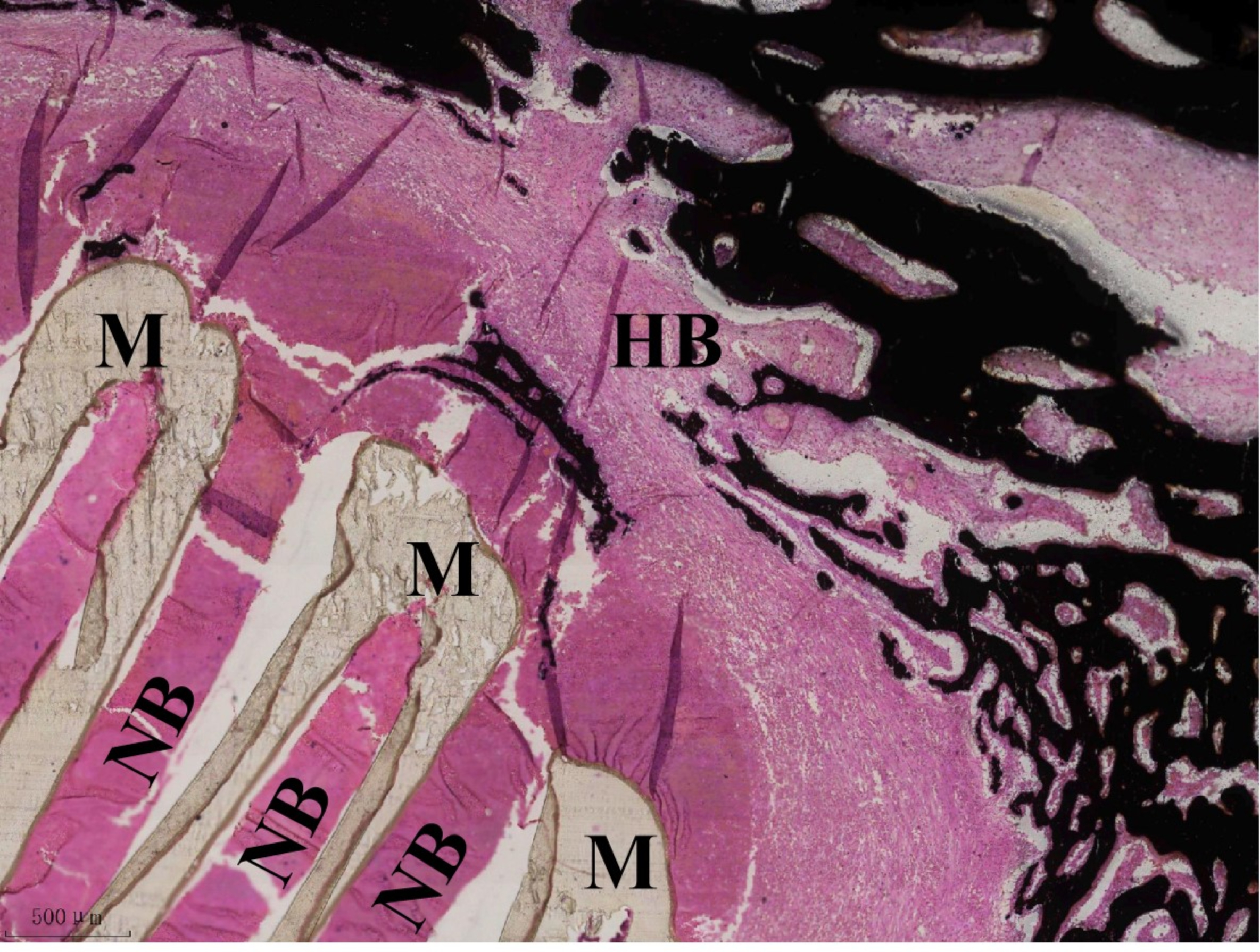

Supplement: Supplementary file 4 — Supplementary Figure 2. [file 41598_2024_56199_MOESM4_ESM.jpg]
